# Establish of an Initial Platinum-Resistance Predictor in High-Grade Serous Ovarian Cancer Patients Regardless of Homologous Recombination Deficiency Status

**DOI:** 10.3389/fonc.2022.847085

**Published:** 2022-03-18

**Authors:** Yongmei Li, Yufei Nie, Hongyan Guo, Hua Guo, Chunfang Ha, Yuan Li

**Affiliations:** ^1^ Department of Gynecology, General Hospital of Ningxia Medical University, Yinchuan, China; ^2^ Department of Obstetrics and Gynecology, Peking University Third Hospital, Beijing, China

**Keywords:** ovarian cancer, initial platinum resistance, machine learning, CCNE1, prognosis, HRD

## Abstract

**Backgrounds:**

Ovarian cancer (OC) is still the leading aggressive and lethal disease of gynecological cancers, and platinum-based regimes are the standard treatments. However, nearly 20%–30% of patients with OC are initial platinum resistant (IPR), and there is a lack of valid tools to predict whether they will be primary platinum resistant or not prior to chemotherapy.

**Methods:**

Transcriptome data from The Cancer Genome Atlas (TCGA) was downloaded as the training data, and transcriptome data of GSE15622, GSE102073, GSE19829, and GSE26712 were retrieved from Gene Expression Omnibus (GEO) as the validation cohorts. Differentially expressed genes (DEGs) were selected between platinum-sensitive and platinum-resistant patients from the training cohort, and multiple machine-learning algorithms [including random forest, XGboost, and least absolute shrinkage and selection operator (LASSO) regression] were utilized to determine the candidate genes from DEGs. Then, we applied logistic regression to establish the IPR signature based on the expression. Finally, comprehensive clinical, genomic, and survival feature were analyzed to understand the application value of the established IPR signature.

**Results:**

A total of 532 DEGs were identified between platinum-resistant and platinum-sensitive samples, and 11 of them were shared by these three-machine learning algorithms and utilized to construct an IPR prediction signature. The area under receiver operating characteristic curve (AUC) was 0.841 and 0.796 in the training and validation cohorts, respectively. Notably, the prediction capacity of this signature was stable and robust regardless of the patients’ homologous recombination deficiency (HRD) and mutation burden status. Meanwhile, the genomic feature was concordant between samples with high- or low-IPR signature, except a significantly higher prevalence of gain at Chr19q.12 (regions including *CCNE1*) in the high-IPR signature samples. The efficacy of prediction of platinum resistance of IPR signature successfully transferred to the precise survival prediction, with the AUC of 0.71, 0.72, and 0.66 to predict 1-, 3-, and 5-year survival, respectively. At last, we found a significantly different tumor-infiltrated lymphocytes feature, including lower abundance of CD4+ naive T cells in the samples with high-IPR signature. A relatively lower tumor immune dysfunction and exclusion (TIDE) value and more sensitivity to multiple therapies including Gefitinib may suggest the potency to transfer from platinum-based therapy to immunotherapy or target therapies in patients with high-IPR signature.

**Conclusion:**

Our study established an IPR signature based on the expression of 11 genes that could stably and robustly distinguish OC patients with IPR and/or poor outcomes, which may guide therapeutic regimes tailoring.

## Introduction

Ovarian cancer (OC) is the seventh most common cancer among women worldwide, and it contributes to the eighth most common cancer-related death (1). The number of new cases and deaths are 239,000 and 152,000 annually, respectively (2). Even though great advancements have been made in the treatment of OC [especially the poly (ADP-ribose) polymerase (PARP) inhibitors], due to disease recurrence and chemoresistance, its 5-year survival rate is still <30% (3). Currently, the standard treatments for high-grade serous ovarian cancer (HGSOC) involve cytoreductive surgery and chemotherapy, and paclitaxel combined with carboplatin is the first choice as the first-line treatment given for six or eight cycles (4). Unfortunately, nearly 20%–30% of patients with HGSOC would have primary resistance to the initial platinum-based therapy (4). Until now, initial platinum resistance (IPR) remains one of the essential obstacles for prolonging the survival of HGSOC patients (5). However, discrimination of platinum-resistant diseases from the sensitive ones is still mainly based on the evaluation of progression-free survival (PFS) of platinum-based regimes. Patients with IPR still have to experience the six cycles of chemotherapy and related adverse events to know whether they are platinum sensitive or platinum resistant. Meanwhile, neoadjuvant chemotherapy (NACT) followed by interval debulking surgery is a promising and standard approach to treat patients with stages IIIC and IV OC who have a low likelihood of optimal cytoreduction (6). Although the surgery results may shed light on the assessment of the platinum sensitivity, NACT itself may increase the risk of secondary platinum resistance (7). Since then, developing a precise model to predict whether the patient will be platinum resistant is highly critical to achieving optimal clinical management of ovarian cancer.

Therefore, previous studies have tried to identify molecular biomarkers associated with platinum resistance and establish prediction models. There are 15%–20% of patients with ovarian cancers harbored germline or somatic alterations in *BRCA1* or *BRCA2* genes. Compared with wild-type ones, patients with *BRCA1/2* alteration had improved efficacy to platinum-based regimes. Furthermore, recently, increasing evidence implicates that “BRCAness” phenotype, including alterations in other homologous recombination repair genes (8) and/or homologous recombination deficiency (HRD) (9), could also confer sensitivity to platinum-based therapy. By utilizing targeted next-generation sequencing, Zheng and her colleagues have established a platinum-sensitivity predictor based on the whole-genome duplication, loss of heterozygosity, and mutational signature (10). Meanwhile, gene expression was also suggested to have close association with the platinum sensitivity in ovarian cancer. For instance, Shannon et al. have identified that the expression signature of *CYTH3*, *GALNT3*, *S100A14*, and *ERI1* has prognostication for platinum sensitivity (11). Non-coding RA, such as miR-23a-3p and miR-206, has also been identified that could serve as effective predictors for platinum sensitivity (12, 13). We have previously developed a genomic signatures-based predictor of IPR in advanced HGSOC patients based on their whole genome and whole exome sequencing data from our local cohort (14); however, the complex testing methods may hinder the predictor from further application in real-world clinical practice. Thus, there are increasing and urgent needs for establishing a robust and convenient predictive tool for the IPR.

In this study, to systematically assess the potential involvement gene signature of IPR, we analyzed the differentially expressed genes by comparing the transcriptome data between platinum-sensitive and platinum-resistant OC samples from The Cancer Genome Atlas (TCGA) database and established an IPR prediction signature by application of multiple machine learning algorithms. Then, we utilized the transcriptome data from Gene Expression Omnibus (GEO) database to validate the prediction capacity of the established model. Furthermore, the clinical, genomic, and prognostic features related to high-IPR signature were also analyzed.

## Materials and Methods

### Data Source

The schematic overview of the study was provided in **Supplementary Figure S1**. The transcriptomic and clinical features of 489 patients with ovarian serous cystadenocarcinoma from the TCGA cohort were derived from the cbioportal website (http://www.cbioportal.org/), setting as the training cohort, and 287 of which had known platinum sensitivity. The GSE15622 dataset, which included 15 ovarian cancer samples with known platinum sensitivity, was downloaded from GEO (https://www.ncbi.nlm.nih.gov/geo/) as the validation cohort of platinum-resistance prediction of IPR signature. Besides, GSE102073 (n=85), GSE19829 (n=28), and GSE26712 (n=195) were retrieved for the GEO as the validation cohort of survival prediction of IPR signature. More details of involved samples in each dataset are provided in **Supplementary Table S1**.

### Differentially Expressed Gene Screening Between Platinum-Sensitive and Platinum-Resistant Samples

Raw gene-level counts were utilized in our analysis. All the data processing and normalization were performed and completed by using the R studio. The gene expression profiling data of samples with RNA expression data were downloaded from the TCGA database. The DEGs between platinum-sensitive and platinum-resistant samples (median value serves as the cutoff) were identified through the “Limma” package from Bioconductor in R language, and p-value <0.01 was set as the threshold for screening the expression difference of DEGs.

### Construction of an IPR Prediction Model Based on Multiple Machine Learning Algorithms

Then, we applied multiple machine learning algorithms, including a boosted decision tree model named eXtreme Gradient Boosting (XGBoost), random forest (RF), and least absolute shrinkage and selection operator (LASSO) to determine the shared key genes from DEGs based on the Venn diagram. To construct the IPR prediction model, logistic regression was further utilized, and the IPR-prediction score formula was established as the expression level of Gene 1 × α_1_ + Gene 2 × α_2_ + Gene 3 × α_3_ +… + Gene n × α_n_, where α_n_ represents the coefficient for the corresponding gene in this model. The median IPR prediction score was responsible for a cutoff value to sort the patients into high- and low-IPR prediction score groups. The survival curves were drawn by using the “survival” package in R studio. The analysis of the area under ROC curve (AUC) was analyzed by the “timeROC” package in R studio. Furthermore, multivariate Cox regression analyses were performed to determine whether the IPR signature is an independent variable for platinum sensitivity. The nomogram calibration plots were constructed by the “rms” package in R, receiver operating characteristic.

### Function Enrichment Analysis

Kyoto Encyclopedia of Genes and Genomes (KEGG) and REACTOME pathway enrichment analysis was carried out for DEGs by clusterProfiler R package. Only the terms with a p-value < 0.05 were considered as significantly enriched in functions of DEGs and pathway analysis.

### Survival Analysis

Kaplan–Meier survival curves were applied to compare the survival data of patients with high- and low-IPR signature. AUC of ROC curve was utilized to evaluate the sensitivity and specificity of survival prediction.

### HRD Scores in the TCGA-OC Cohort

HRD score or the genomic instability score was calculated as a sum of loss of heterozygosity (LOH), large-scale transition (LST), and telomeric allelic imbalance (TAI) in each sample. The pre-calculated HRD results of 169 patients from the TCGA-OC cohort were adopted from Knijnenburg et al., which were based on the genotyping array data from TCGA (15). The number of segment LOH were generated by TCGA Network Aneuploidy Analysis Working Groups using ABSOLUTE algorithm (16, 17). The chromosomal breaks between adjacent regions of at least 10 Mb were defined as LST, and the number of LSTs was estimated for each chromosome arm independently (18). The number of TAI was calculated of the number of regions that extend to one of the sub-telomeres but do not cross the centromere (19). The threshold for HRD score was 42, and samples with HRD scores below 42 were considered as homologous recombination proficiency (HRP).

### Tumor-Infiltrating Immune Cells and Tumor Immune Dysfunction and Exclusion Analysis

The abundance of immune cells was analyzed using XCELL online software (https://xcell.ucsf.edu/). mRNA expression data of samples with high- and low-IPR signature were uploaded to the website to predict the proportion of immune cell types. Similarly, to predict the response to immunotherapy, the RNA-seq data of samples with high- and low-IPR signature from the training cohort were used to analysis the tumor immune dysfunction and exclusion (TIDE) value (20).

### Drug-Sensitivity Analysis

Candidate drug sensitivity of OC patients with high- or low-IPR signature in the training cohort was analyzed by the Genomics of Drug Sensitivity in Cancer database (GDSC; https://www.cancerrxgene.org/), and the half-maximal inhibitory concentration (IC_50_) was calculated through the “pRRophetic” (21). Drugs with significantly lower IC_50_ (p < 0.05) were considered as sensitive.

### Statistical Analyses

Log-rank test in each data set was applied to analyze the difference in survival. Chi-square, Fisher test, and Wilcoxon rank test statistical analyses were all performed by using R studio (v. 3.4.3, https://rstudio.com/). A (adjusted) p-value <0.05 was considered statistically significant.

## Results

### Establishment of an IPR Signature

**Figure 1:**
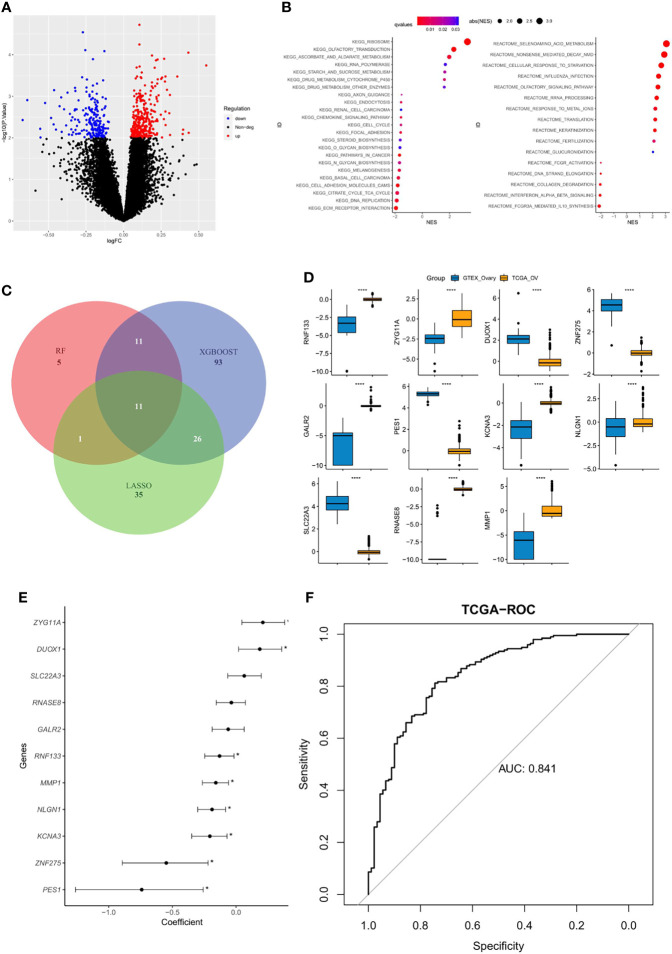
Construction of an IPR signature in the training cohort (TCGA-OV). **(A)** Differentially expressed genes (DEGs) between platinum-sensitive and platinum-resistant samples. **(B)** Enriched functional pathway of platinum sensitivity-related DEGs. **(C)** Venn diagram revealed the overlapping candidate genes of XGBoost, random forest (RF), and least absolute shrinkage and selection operator (LASSO). **(D)** The expression level of the 11 candidate DEGs between normal ovarian cells (GTEX) and tumor tissues (TCGA), ^****^ *p* < 0.00001. **(E)** Correlation between each candidate DEG and the platinum sensitivity, ^*^ *p* < 0.05. **(F)** The prediction efficacy of the established model in the training cohort.

The DEGs between platinum-sensitive and platinum-resistant OC samples were analyzed, and a total of 532 DEGs were identified, including 332 significantly upregulated and 200 downregulated genes (**Figure 1A**). These DEGs were enriched in multiple pathways, including olfactory transduction, RNA polymerase, cytochrome P450, chemokine signaling pathway, and cell cycle (**Figure 1B**). Then, 27, 141, and 73 candidate DEGs were further selected by RF, XGBoost, and LASSO, respectively, 11 of which were shared by these three machine learning algorithms (**Figure 1C**). The expression of overlapped 11 candidate genes significantly differed between normal ovarian cells and tumor samples (**Figure 1D**), and eight of them were significantly correlated with platinum sensitivity (p<0.05, **Figure 1E**). Next, by utilizing multivariate logistic regression analysis, an IPR prediction model was established, and the formula was as follow: IPR predictor score = RNF133*−2.33 + *ZYG11A* * 0.380 + *DUOX1* * 0.313 + ZNF275* −1.769 + GALR2*1.680 + PES1*−0.618 +KCNA3*−1.691 +NLGN1*−0.752 + SLC22A3*1.769 + RNASE8*−1.294 + MMP1*−0.242. This IPR prediction model reached an AUC value of 0.841 to predict IPR in the training cohort (**Figure 1F**).

### Validation of the Established IPR Signature

**Figure 2:**
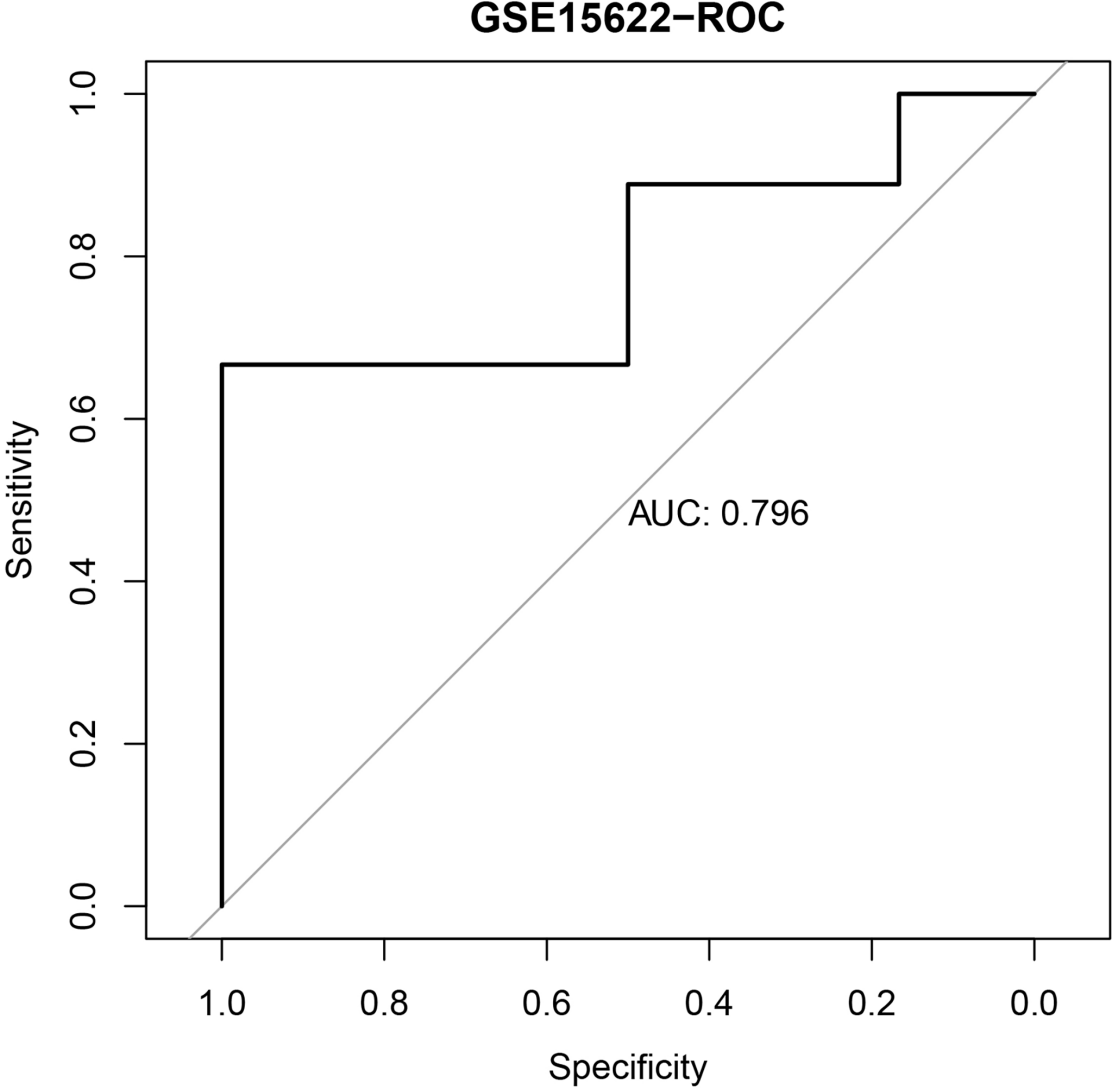
The ROC curve of the IPR signature in prediction the platinum resistance in the validation cohort (GSE15622).

We further validated the established IPR prediction score model in the independent validation cohort (GSE15622). The AUC value for predicting the IPR was 0.796, which indicated the satisfactory performance of the IPR prediction score model (**Figure 2**).

### Comparison With HRD Score

**Figure 3:**
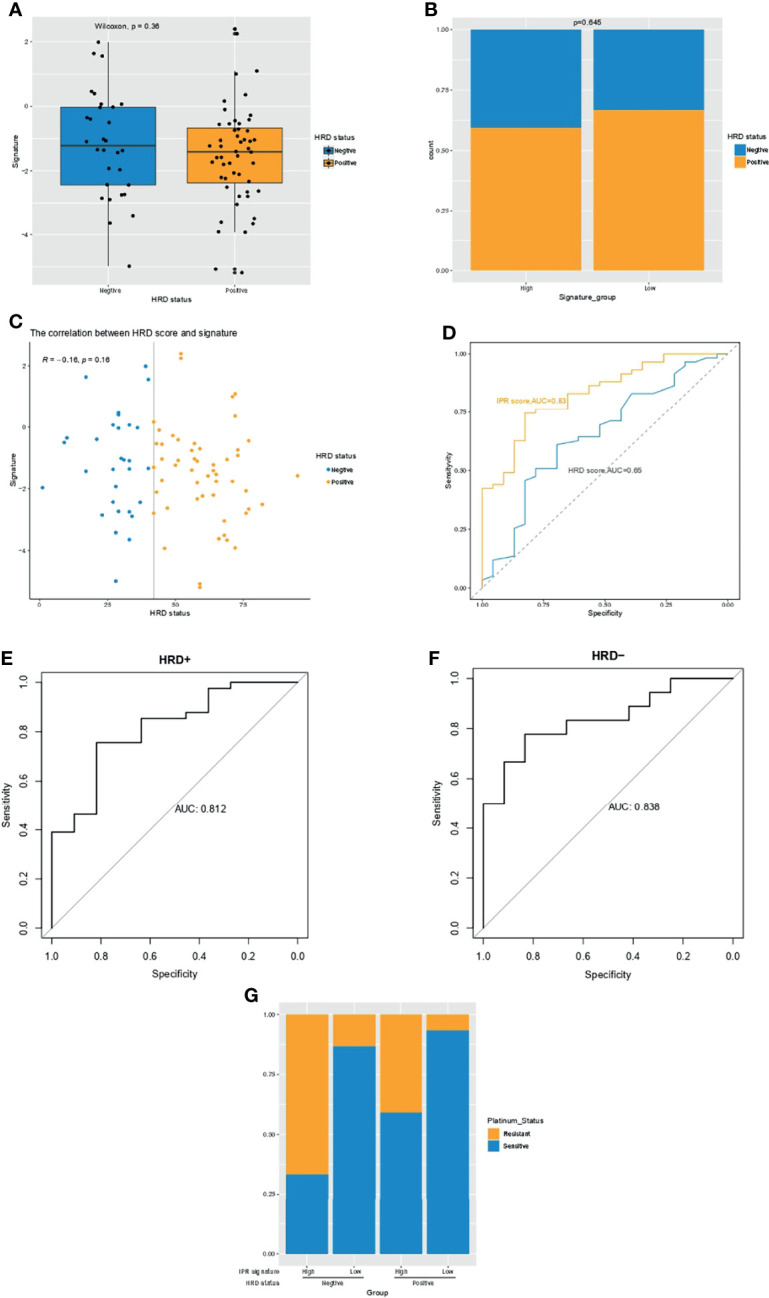
The relationship between HRD and the IPR signature in the training cohort. **(A)** The difference in the IPR signature score between OC samples with HRD and HRP. **(B)** There was no significance in the prevalence of HRD between samples with high- or low-IPR signature. **(C)** Correlation between IPR signature score and HRD score. **(D)** Comparison of the IPR prediction capacity between IPR signature and HRD score. The prediction efficacy was stable in both the HRD **(E)** and HRP **(F)** samples. **(G)** The prevalence of platinum resistance in samples with different HRD and IPR signature. HRD, homologous recombination deficiency; HRP, homologous recombination proficiency; IPR, initial platinum resistance.

Previous studies have suggested HRD score as a promising predictor for platinum sensitivity; thus, we investigated the relationship between HRD score and the constructed IPR signature. There was neither significant difference in the IRP signature score between HRD and HRP samples nor in the HRD score between samples with high- or low-IPR signature (**Figures 3A, B**). HRD score had no significant correlation with IPR signature score (R=−0.16, p=0.16, **Figure 3C**). IPR signature had superior efficacy than HRD score in predicting platinum resistance (AUC, 0.83 vs. 0.65, p=0.035, **Figure 3D**). Meanwhile, it was noteworthy that the prediction capacity was stable regardless of the HRD status (**Figures 3E, F**). Groups with HRP and high-IPR signature had a significantly higher prevalence of platinum-resistant samples (HRP-IPR^high^ versus other groups, 66.7% vs. 19.40%, p<0.001, **Figure 3G**).

### Mutation Count and the IPR Signature

**Figure 4:**
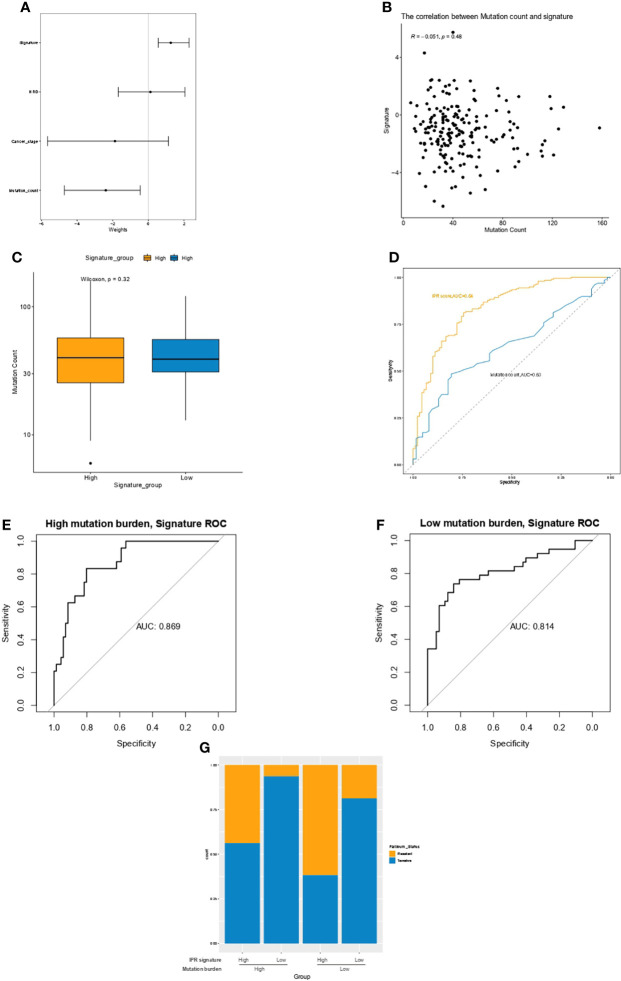
The relationship between mutation count and the IPR signature in the training cohort. **(A)** Multivariate Cox regression analysis identified the mutation count and IPR signature were the two independent variable for the platinum sensitivity. **(B)** There was no significant correlation between IPR signature score and mutation count. **(C)** The difference in the IPR signature score between OV samples with HRD and HRP. There was no significance in the mutation counts between samples with high- or low-IPR signature. **(D)** IPR signature outperformed than mutation counts in predicting the IPR. The prediction efficacy was stable in both the high- **(E)** and low-mutation burden **(F)** samples. **(G)** The prevalence of platinum resistance in samples with different mutation counts and IPR signature. IPR, initial platinum resistance.

Multivariate Cox regression analysis identified the mutation count, and IPR signature were the only two independent variables for the platinum sensitivity in the training cohort (**Figure 4A**). Then, we analyzed the relationship between mutation counts and IPR signature. The results revealed no correlation between IPR signature score and mutation count (R=−0.051, p=0.48, **Figure 4B**). Similarly, the mutation counts levels were not significantly different between OC samples with high- or low-IPR signature (**Figure 4C**). Although these two variables were related to platinum resistance, IPR signature outperformed mutation counts in predicting the IPR (AUC, 0.84 vs. 0.63, p < 0.001, **Figure 4D**). On the mutation burden stratification, the prediction efficacy was also stable in both the high- (AUC=0.869) and low-mutation burden (AUC=0.814) samples (**Figures 4E, F**). The prevalence of platinum-resistance samples was significantly enriched in the samples with low mutation burden but high-IPR signature (TMB^low^ IPR^high^ versus other groups, 61.70% vs. 23.08%, p<0.0001, **Figure 4G**).

### Comprehensive Analysis of the Difference in the Genomic Feature Between Samples With High- or Low-IPR Signature

**Figure 5:**
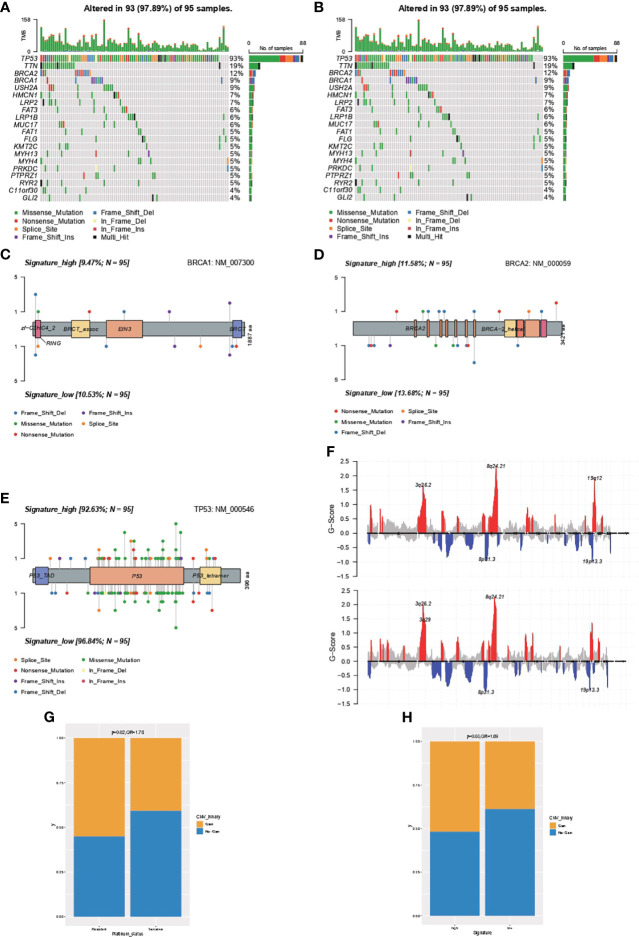
Comprehensive analysis on the difference in the genomic feature between samples with high- or low-IPR signature. Oncoprint was generated for the most prevalent altered genes in samples with high- **(A)** or low-IPR **(B)** signature. The details in the distribution and prevalence of *BRAC1* **(C)**, *BRCA2* **(D)**, and *TP53* **(E)** are shown in the lollipop plots. Upper: high-IPR signature; below: low-IPR signature. **(F)** Copy number changes in samples with high- (upper) or low-IPR (below) signature. A higher prevalence of gain at 19q12 was identified both in the platinum-resistant **(G)** and high-IPR signature **(H)** samples.

The genomic feature of the most frequently altered genes was in concordance between samples with high- or low-IPR signature, with a leading prevalence of alterations in *TP53*, *TTN*, *BRCA2*, *BRAC1*, and *USH2A* (**Figures 5A, B** **)**. Furthermore, as there was no gene with significantly distinct prevalence, we specifically analyzed the distribution of alterations in *BRCA1* (**Figure 5C**), *BRCA2* (**Figure 5D**), and *TP53* (**Figure 5E**) but found no significant difference as well. In addition, we analyzed the copy number changes in these two groups (**Figure 5F**) and identified a higher prevalence of gain at Chr19.12 in samples with high-IPR signature. Gain at Chr19.12 was more prevalent in platinum-resistant samples (odds ratio, OR=1.78, p=0.02, **Figure 5G**) and in samples with high-IPR signature (OR=1.69, p=0.03, **Figure 5H**).

### IPR Signature Had Capacity in Survival Prediction in the Training Cohort

**Figure 6:**
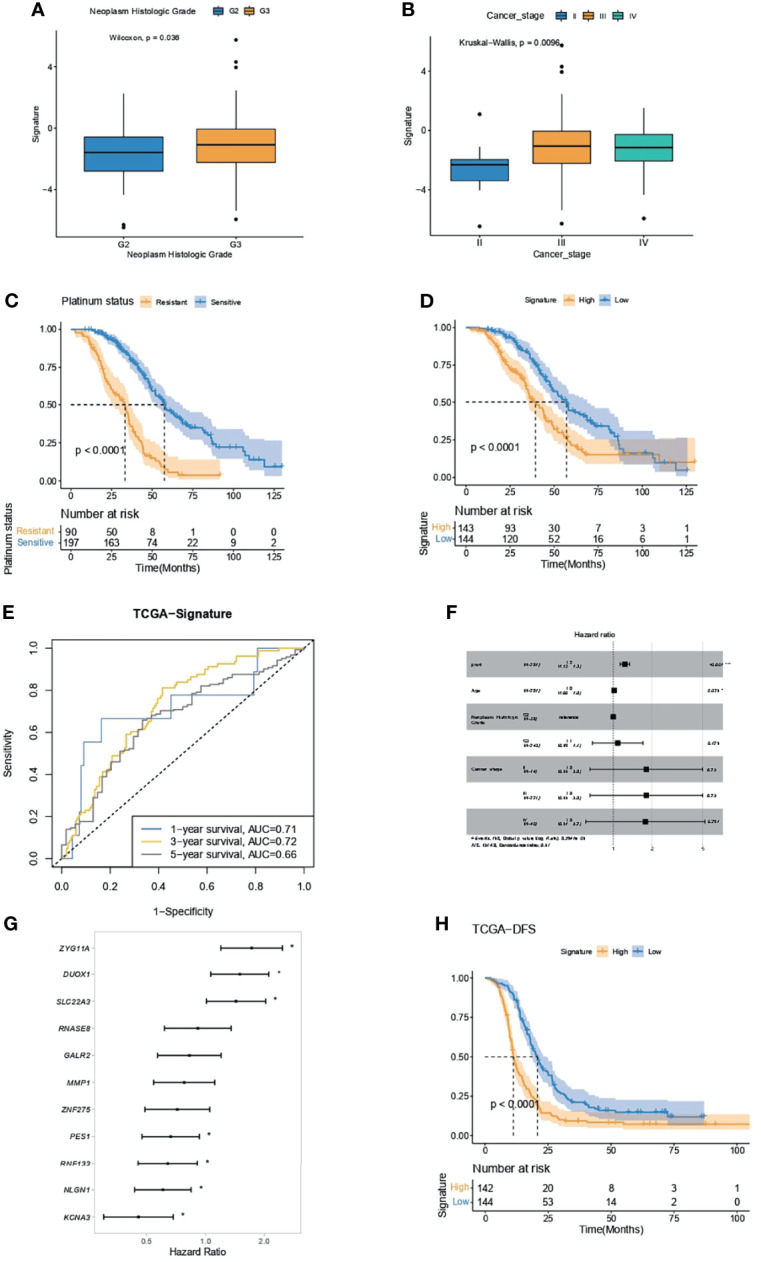
IPR signature had the capacity in survival prediction in the training cohort. **(A)** The difference in IPR signature score between samples with G2 and G3 neoplasm histological grade. **(B)** The difference in IPR signature score between samples with different cancer stages; Kaplan–Meier analysis showed a significantly inferior overall survival in platinum-resistant ovarian cancer patients **(C)** and high-IPR signature patients **(D)**. **(E)** The ROC curve of the IPR signature in predicting the 1-, 3-, and 5-year survival in the training cohort. **(F)** Multivariate Cox regression analysis identified that the IPR signature and age were the two independent variable for the overall survival. **(G)** Forest plot of the hazard ratio for the association of each gene related to IPR signature score with overall survival. **(H)** Kaplan–Meier analysis of the disease-free survival (DFS) of patients with different IPR signature score. *p < 0.05.

OC patients with higher neoplasm histologic grade (**Figure 6A**) or cancer stage (**Figure 6B**) had significantly higher IPR signature scores. As OC patients who were platinum resistant had significantly inferior overall survivals (OS, **Figure 6C**), we analyzed the difference in survival between patients with high- or low-IPR signature. Likewise, patients with high-IPR signature had significantly worse OS than those with low-IPR signature (**Figure 6D**). The AUC for IPR signature to predict 1-, 3-, and 5-year survival was 0.71, 0.72, and 0.66, respectively (**Figure 6E**). Multivariate Cox regression analysis identified the IPR signature as the independent variable for predicting overall survival (HR=1.2, 95% CI 1.13–1.3, p<0.001, **Figure 6F**). Among those 11 genes, three (*ZYG11A*, *DUOX1*, and *SLC22A3*) were significantly associated with worse survival, and four (*PES1*, *RNF133*, *NLGN1*, and *KCNA3*) were significantly associated with better survival (p<0.05, **Figure 6G**). Additionally, patients with high-IPR signature also had significantly inferior disease-free survival (DFS) than those with low-IPR signature (**Figure 6H**).

### Correlation Between IPR Signature and Clinical Feature in Survival Prediction

**Figure 7:**
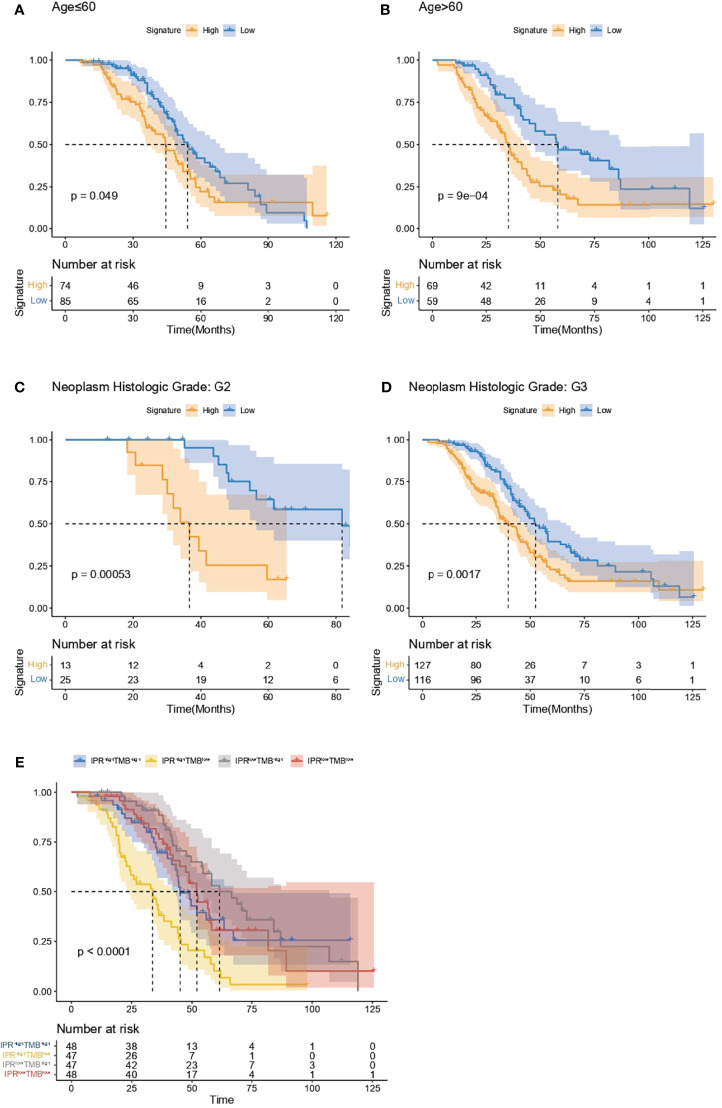
Analysis of the survival prediction of IPR signature in patients with different clinical feature. Kaplan–Meier analysis of the overall survival of patients diagnosed below **(A)** and beyond 60 years old **(B)**, neoplasm histological grade of G2 **(C)** and G3 **(D)**. **(E)** Kaplan–Meier analysis of the overall survival on the stratification of mutation burden and IPR signature.

Regardless of the patients’ age (below or beyond 60 years old), the IPR signature could significantly distinguish patients with worse overall survival (**Figures 7A, B**). On neoplasm histological grade stratification, high IPR signature score was associated with significantly shorter overall survival in both G2 and G3 disease (**Figures 7C, D**). Because the IPR signature and mutation counts were the only two independent variables related to platinum resistance, we analyzed the survival difference on the stratification of mutation counts and IPR signature (**Figure 7E**). Patients with low-TMB but high-IPR signature had the most inferior OS than other groups (median OS for TMB^low^IPR^high^, TMB^high^IPR^high^, TMB^low^IPR^low^, TMB^high^IPR^low^: 32.03, 33.28, 58.08, and 55.88 months, respectively).

### Validation of the Capacity of IPR Signature in Survival Prediction

**Figure 8:**
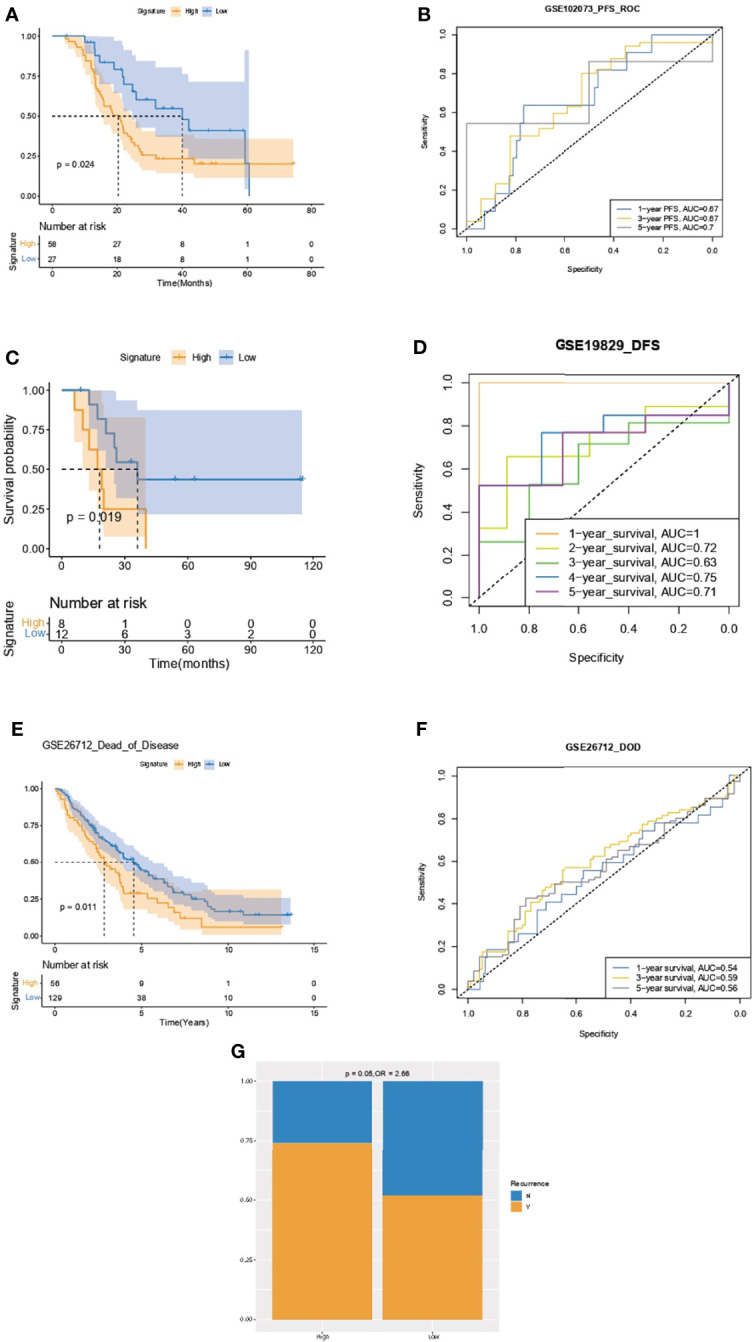
Validation of the capacity of IPR signature in survival prediction. Kaplan–Meier analysis and ROC curve of the IPR signature in prediction the PFS in the GSE102073 **(A, B)**, DFS in GSE19829 **(C, D)**, and time to DOD in GSE26712 **(E, F)**. **(G)** The distribution of patients with disease recurrence in the high- or low-IPR signature. Ovarian cancer patients were divided into high- and low-IRP signature group according to the optimal cutoff value in each cohort. PFS, progression-free survival; DFS, disease-free survival; DOD, dead of disease.

Kaplan–Meier analysis and ROC curve supported that the IPR signature could successfully distinguish patients with inferior PFS in the GSE102073, with the AUCs of 0.67, 0.67, and 0.7 to predict 1-, 3-, and 5-year PFS (**Figures 8A, B**). It also robustly predicted the DFS in GSE19829 (**Figures 8C, D**) and the dead of disease (DOD) time in GSE26712 (**Figures 8E, F**). Furthermore, we analyzed the relationship between disease recurrence and the IPR signature and found significantly higher prevalence of disease recurrence in patients with high-IPR signature (74% vs. 51%, p=0.05, **Figure 8G**).

### Estimation of the Associations Between Immune Characteristics, Potential Therapy, and the IPR Signature

**Figure 9:**
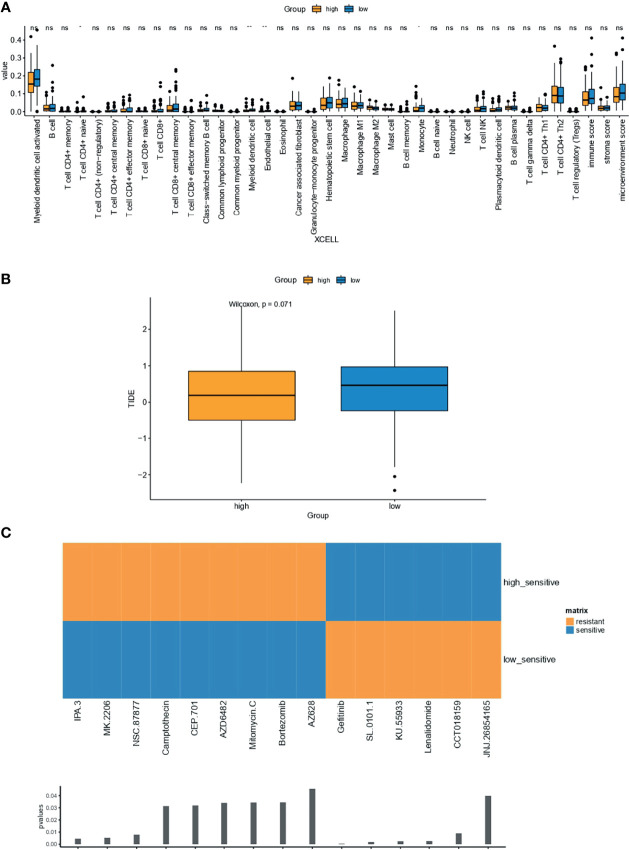
Estimation of the associations between immune characteristics, potential therapy, and the IPR signature. The difference in the abundance of tumor-infiltrating immune cells were analyzed by XCELL **(A)** in the training cohort, independently. **(B)** The difference in TIDE value between high- and low-IRP signature group. **(C)** Drugs with significantly different sensitivity were analyzed by utilizing the GDSC dataset. TIDE, tumor immune dysfunction and exclusion. *p < 0.05; **p < 0.01; ns, not significant.

Compared with OC samples with low-IPR signature, samples with high-IPR signature were presented with a significantly lower abundance of CD4+ naive T cells, myeloid dendritic cells, endothelial cells, and monocytes (**Figure 9A**). Meanwhile, although without statistical significance, samples with high-IPR signature had relatively lower TIDE value, which may indicate a relatively higher potency to respond to immune checkpoint blockade (ICB, **Figure 9B**). Furthermore, samples with low-IPR signature were more sensitive to therapies, including IPA.3, MK2206, NSC.87877, and other regimes (CEP.101, AZD6482, Mitomycin.C, Bortezomib, AZ628); on the contrary, samples with high-IPR signature were significantly more sensitive to Gefitinib and other therapies including SL.0101.1, KU55933, Lenalidamoide, CCT018159, and JNJ.26854165 (**Figure 9C**).

## Discussion

Prognostication of platinum resistance prior to chemotherapy in patients with ovarian cancer is still an unmet problem. Using the TCGA database as the training cohort, we have identified a total of 532 platinum-sensitivity-related DEGs and finally found 11 as the candidate DEGs by three machine learning algorithms including XGBoost, RF, and LASSO. Even though each of these algorithms has been widely applied and owns stable and accurate performance in constructing prediction model, a combination of them would provide more solid results. Meanwhile, downsizing of the candidate genes by combining the three algorithms would provide more convenience for the further clinical application of the established prediction model. The platinum-resistance prediction capacity of the established model reached an AUC of 0.841 and 0.796 in the training and validation cohorts, respectively, indicating that it was a robust model to prognosticate platinum resistance for patients with ovarian cancer. Meanwhile, its prognosticate power was stable regardless of the HRD status; it would, therefore, be tempting to suggest that the established IPR signature could effectively distinguish platinum-resistant patients from HRD-positive groups.

Among the 11 selected genes, notably, three of them including *SLC22A3*, *DUOX1*, and *MMP1* were all related to mitochondrial-related metabolism pathways. This feature was in concordance with the results from previous studies that mitochondrial dynamics played a pivotal role in the chemoresistance of OC patients (22). Platinum agents could bind to DNA and induce DNA damage, resulting in mitochondria-mediated apoptosis, and previous studies have found that there were notable difference in both mitochondrial contents and mitochondria-related reactive oxygen species (ROS) production between ovarian cells sensitive and resistant to cisplatin (23). Meanwhile, Han and his collogue found that tumor environment, especially hypoxia status, could induce platinum resistance by triggering ROS production and thus causing mitochondria fission (24). *ZYG11A*, a member of *ZYG11* family, has been identified to be involved in the regulation of cell cycle and division, and its expression in ovarian cancer was correlated with tumor stage (25). Whether it serves as an oncogene or tumor suppressor gene in tumor development is still controversial, but limited evidence suggests that it functions as an oncogene by interacting with CCEN1 in non-small lung cancer (26). We found that it was associated with high hazard ratio to prognosticate OS, suggesting its role as an oncogene in OC. *PES1*, involved in the pre-ribosomal RNA processing, is an oncogene that promotes the carcinogenesis and development of different types of cancers (27). Although evidence on its role in OC was still poor, an early study suggested that it could affect the progression by regulation the ER in OC (28). *KCNA3* has been identified as a key immune-related gene in ovarian cancer, and moreover, its overexpression was associated with disease stage and superior survival (29). Other key genes, including *NLGN1* (30) and *GALR2* (31), were identified in the cancers, but their roles in ovarian cancer, especially in platinum sensitivity were unreported before. We identified that *NLGN1* was significantly associated with the platinum sensitivity and survival of OC patients.

The genomic feature was in concordance between patients with high- or low-IPR signature, including genetic alterations, TMB, and HRD. To date, even though the association between HRD scores and platinum agents’ sensitivity has been extensively studied in various cancers, it is still full of controversy. For instance, in breast cancer, TNT (32) and GeparSixto trial (33) presented the distinct role of HRD in predicting the response of platinum agents: in TNT trial, HRD status failed to stratify advanced breast cancer patients who benefitted more from carboplatin than docetaxel; however, patients with HRD from GeparSixto trial had higher pathological complete response rates from platinum-based than those of non-platinum-based therapy. For ovarian cancer, multiple trials, for instance, PAOLA-1 and PRIMA, all demonstrated that over half of platinum-sensitive HGSOC were positive of HRD (34), and OC patients who were platinum sensitive had higher HRD scores than those who were platinum resistant (9). In this study, our novel IPR signature significantly outperformed the HRD score in predicting the platinum sensitivity (AUC value, 0.83 vs. 0.65). Moreover, the accuracy of the IPR signature was stable both in HRD and non-HRD OC patients, which would be of clinical value to recognize potential IPR patients who have HRD. On the other hand, TMB is a novel and Food and Drug Administration (FDA)-approved biomarker-associated higher efficacy of immunotherapy in pan-cancer (35). However, as the response rates of immunotherapy were extensively poor in OC, the role of TMB is also being continuously overlooked. The TMB value of OC is medium among pan-cancers, and <5% of OC cases have TMB values beyond the threshold (10 mut/Mb) (36). In other cancer types, evidence has suggested that TMB, a biomarker of genomic instability, presented with predictive value in platinum-based chemotherapy (37). Interestingly, through multivariate Cox regression analysis, we found that TMB (or mutation counts) could serve as an independent factor predicting the platinum sensitivity, which is contradicted with the results of our previous study in which we analyzed 99 local HGSOC patients’ tumor samples but found that TMB alone has limited capacity in predicting IPR (AUC=0.5433) (38). Furthermore, the IPR model was stable and robust no matter whether patients presented with TMB-high phenotype or not.

Additionally, a higher prevalence of gain 19q12 in patients with high-IPR score was identified, namely, locus encoded cyclin E1 (CCNE1) and URI1, and has been previously identified to be associated with chemoresistance in ovarian cancer patients (39–41). Noteworthy, the gain at 19q12 was associated with genomic instability and poorer survival in ovarian cancer patients (42), and the amplification of *CCNE1* has been found as a discriminating biomarker for first-line platinum-based chemotherapy. *CCNE1*, which functions as the regulator for the transition from G1 to S phase and determines cell division, mainly was amplificated in the HR-proficient ovarian cancer cells (43). As platinum agents mainly function by causing intra- and inter-strand crosslinks and further inducing cell cycle arrest and cell death, the mutual exclusivity of HRD and *CCNE1* amplification contributes to accurate DNA repair in cell, which in turn removes platinum-induced DNA damage and causes platinum resistance (41). Furthermore, this region could be added to the lists of inquiries for drug development that might be currently underappreciated.

Furthermore, we found a higher sensitivity of Gefitinib in patients with high-IPR signature. Gefitinib is a selective tyrosine kinase receptor inhibitor targeted at epidermal growth factor receptor (EGFR) alterations and has been approved in the treatment of patients with non-small cell lung cancer (44), and *in vitro* research found that it had synergistic effects with cisplatin (45). EGFR and phosphorylated EGFR could be widely detected in the ovarian cancer samples, and Gefitinib monotherapy could decrease their levels. Unfortunately, Gefitinib monotherapy exhibited limited clinical efficacy (with a response rate of 0%–9%) in ovarian cancer, but several combinatorial strategies have shown promising efficacy (46). On the other hand, even though ICBs have made great advances in multiple types of cancers, the efficacy of ICBs (monotherapy or in combination with chemotherapy) in OC remains poor (response rate below 10%) (47). Analysis of the tumor microenvironment of OC, especially the T-cell immunity, provided unique insights into the mechanism of resistance to ICBs (48). TIDE is a more effective algorithm in predicting the tumor immune evasion and response of ICB than the widely applied prediction biomarkers, such as PD-L1 expression level and TMB (49). OC patients with high-IPR signature exhibited relatively lower TIDE value, supporting a lesser T-cell dysfunction microenvironment and a better efficacy to ICBs.

A limitation of this study is that survival and platinum-resistance prediction of the established IPR prediction score was mainly based on the public databases, which merited further validation in the real clinical samples. Further validation study utilizing local patients’ samples and real-time PCR is needed to validate and simplify the testing method of the established predictor. Meanwhile, more preclinical experiments are needed to explore the efficacy of the involved therapeutic regimes, such as ICB, Gefitinib, or therapy targeted at *CCEN1*.

In conclusion, the novel constructed IPR prediction score had the good sensitivity and specificity as a prognostic predictor for platinum sensitivity and could also distinguish OC patients with inferior survivals.

## Data Availability Statement

The original contributions presented in the study are included in the article/**Supplementary Material**. Further inquiries can be directed to the corresponding authors.

## Author Contributions

CH; YuL proposed and designed the study; YoL; YN collected the raw data and conducted data analysis; YN; HoG mainly validated the results; YoL, YN, HoG, HuG, CH; YuL wrote the original manuscript; CH; YuL revised the manuscript draft; All authors agreed this manuscript to be published.

## Funding

This study was funded by the National Natural Science Foundation of China No. 82102769.

## Conflict of Interest

The authors declare that the research was conducted in the absence of any commercial or financial relationships that could be construed as a potential conflict of interest.

## Publisher’s Note

All claims expressed in this article are solely those of the authors and do not necessarily represent those of their affiliated organizations, or those of the publisher, the editors and the reviewers. Any product that may be evaluated in this article, or claim that may be made by its manufacturer, is not guaranteed or endorsed by the publisher.
