# Supplementary figures and images for: Establish of an Initial Platinum-Resistance Predictor in High-Grade Serous Ovarian Cancer Patients Regardless of Homologous Recombination Deficiency Status
Source: Front Oncol. 2022 Mar 18;12:847085. doi: 10.3389/fonc.2022.847085 (PMC8971787; doi:10.3389/fonc.2022.847085)

Model Construction

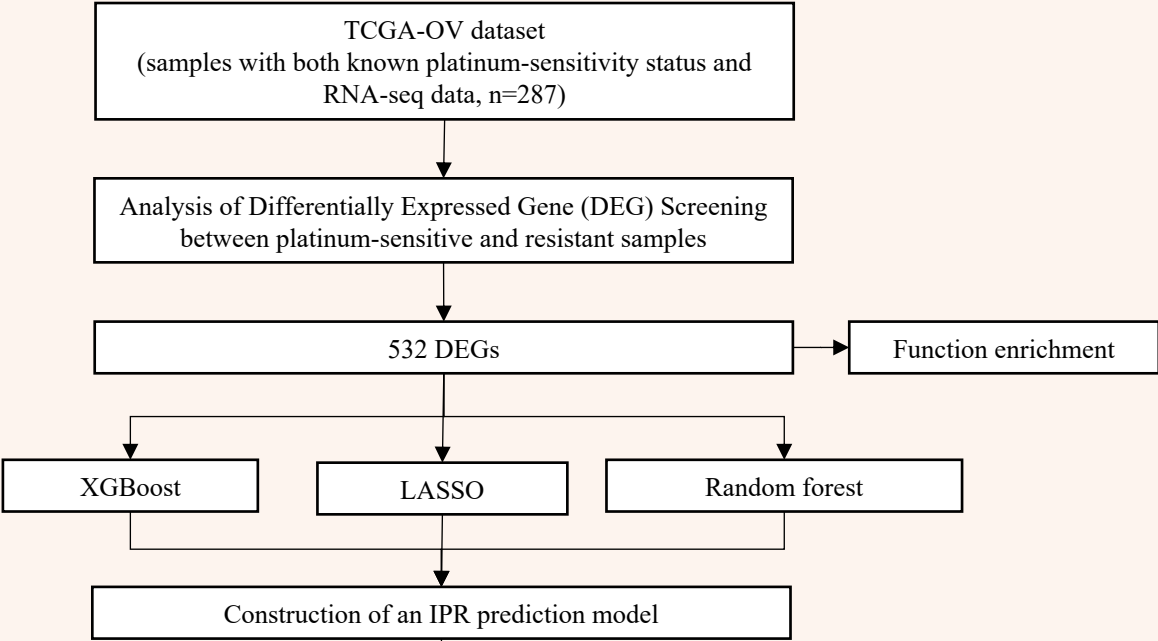

Validation

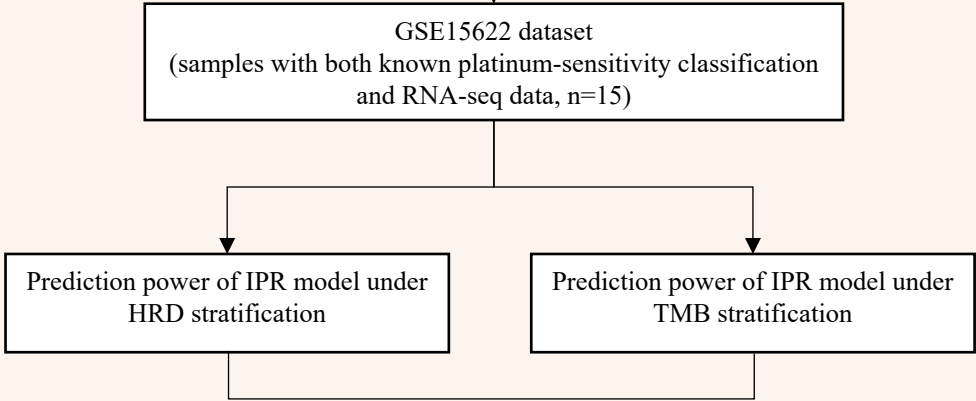

Exploration and Application

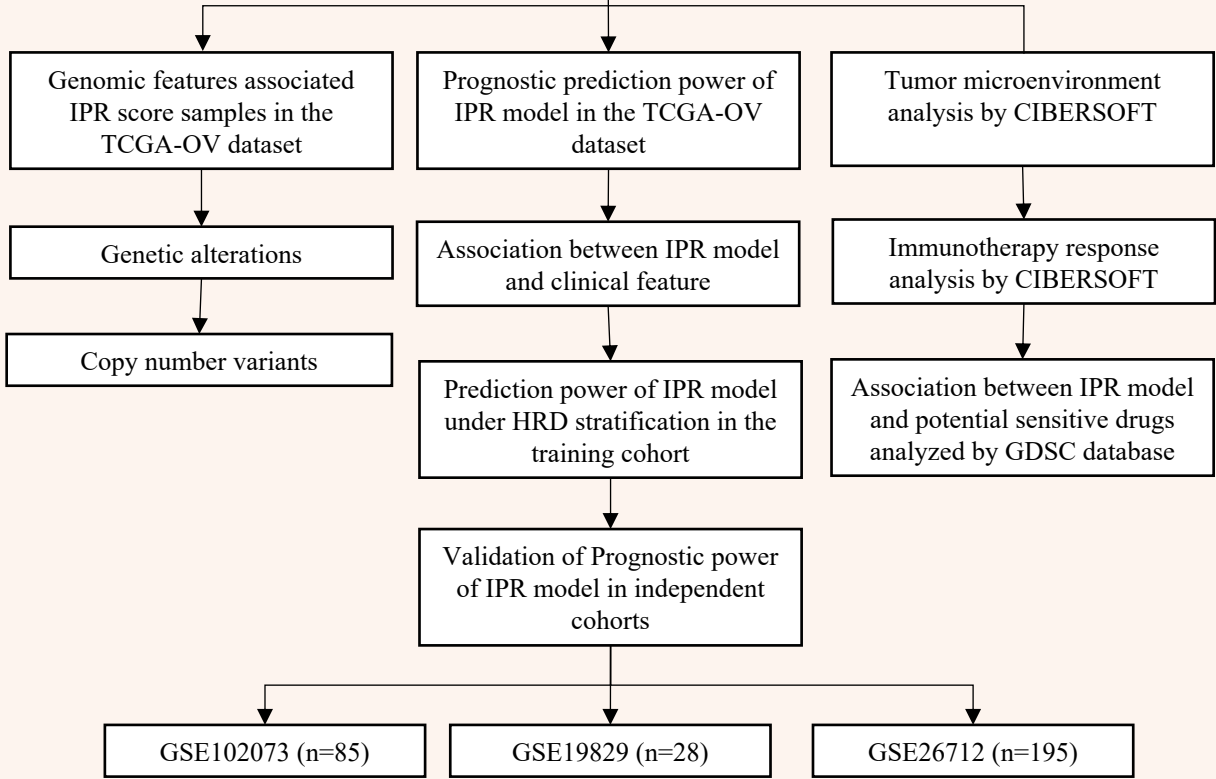

Supplement: Supplementary file 1 [file DataSheet_1.pdf]
