# Supplementary material for: Establish of an Initial Platinum-Resistance Predictor in High-Grade Serous Ovarian Cancer Patients Regardless of Homologous Recombination Deficiency Status
Source: Front Oncol. 2022 Mar 18;12:847085. doi: 10.3389/fonc.2022.847085 (PMC8971787; doi:10.3389/fonc.2022.847085)
Supplement: Supplementary file 2 [file DataSheet_2.pdf]

Dataset	TCGA		GSE15622			GSE102073		GSE26712		GSE19829	
Sample type	Frozen biospecimens		Fresh or frozen biospecimens			Frozen tissue tumor blocks		FFPE sample		FFPE sample	
Sequencing method	Next-generation sequencing and microarry		Microarray			Next-generation sequencing		Microarray		Microarray	
Total samplel size with expression data utilized		489			15*		85		195		28
Age（median, range）		59[30,87]			NA		59[42,79]		NA		68[47,89]
Clinical_stage	I	7		I	NA	I	1	I	NA	I	0
	II	24		II	NA	II	3	II	NA	II	2
	III	381		III	NA	III	56	III	NA	III	22
	IV	79		IV	NA	IV	25	IV	NA	IV	4
Platinum_status	Sensitive	197	latinum_status	Sensitive	9						
	Resistant	90		Resistant	6						

*Samples treated with paclitaxel monotherapy were not included in this analysis
